# Effectiveness of Large-Scale Preparedness Training on Electrocardiogram for Medical, Surgical, and Pre-clinical Doctors: A Need-Based Initiative for COVID-19 Patient Care

**DOI:** 10.7759/cureus.22011

**Published:** 2022-02-08

**Authors:** Arun Varghese, Minakshi Dhar, Shalinee Rao, Rohit Raina, Sunita K Mittal, Barun Kumar, Nowneet Bhat

**Affiliations:** 1 College of Nursing, All India Institute of Medical Sciences Rishikesh, Rishikesh, IND; 2 Internal Medicine, All India Institute of Medical Sciences Rishikesh, Rishikesh, IND; 3 Pathology, All India Institute of Medical Sciences Rishikesh, Rishikesh, IND; 4 Physiology, All India Institute of Medical Sciences Rishikesh, Rishikesh, IND; 5 Cardiology, All India Institute of Medical Sciences Rishikesh, Rishikesh, IND; 6 Pediatric Medicine, All India Institute of Medical Sciences Rishikesh, Rishikesh, IND

**Keywords:** skills, training, electrocardiogram, effectiveness, covid-19

## Abstract

Introduction: One of the competencies expected of all doctors posted in coronavirus disease 2019 (COVID-19) wards, is ECG rhythm identification, interpretation, and intervention for immediate management of patients. This study was undertaken to evaluate the effectiveness of the ECG training module as a component of preparedness training to combat COVID-19.

Methodology: This was a cross-sectional study conducted during training on ECG rhythm identification, interpretation, its management in COVID-19 patients. Study participants included faculty, senior residents, junior residents, and interns of medical, surgical, and paraclinical disciplines. The training session included one hour of didactic lecture and one and half hours of interactive session during which case scenarios were discussed. An objective assessment was conducted through pre-test and post-test. Mean of pre and post-test scores were compared using paired t-test for evaluating statistical significance. Feedback was also taken from participants.

Results: Out of the 800 participants who gave consent, only 682 who completed both pre and post-test were included in the final analysis. Mean pre-test and post-test scores were 9.29/15 (61.9%) and 11.63/15 (77.5%), respectively, with a mean improvement of +2.34/15 (+15.6%). Of the participants, 38.6% obtained low scores in pre-test and 82% of respondents agreed that knowledge and skills gained from training would be useful in providing patient care.

Conclusion: Low baseline knowledge on ECG highlights the need for re-training doctors posted in COVID-19 care on cardiac rhythm identification and interpretation. Interactive training is effective in improving ECG interpretative skills among doctors across disciplines and is the appropriate method to retrain/reskill, especially for large-scale capacity building.

## Introduction

“Necessity is the mother of Invention” is an old saying and still holds good. Coronavirus disease 2019 (COVID-19) has created unimaginable situations with specialists becoming the generalists and vice versa. COVID-19 rapidly engulfed the entire world and compelled out-of-box solutions to various problems posed during the pandemic. There was an increased demand for healthcare workers (HCWs) to manage moderately and critically ill patients. The daily increase in cases demanded expansion of critical care areas with urgent functionality. Clinicians and nurses from various specialities were posted in COVID-19 care wards. Therefore, a major challenge was to ensure these health professionals involved are well versed with the skills required to manage COVID-19 patients. In this direction, our hospital scheduled several skill-based training for healthcare professionals as per their scope of work. One such important training was on ECG cardiac rhythm identification, interpretation, and basic knowledge for controlling the rhythm before the experts could arrive.

Performing, reading, and interpreting ECG is an essential competency and a part of medical education and training but with time and specialization in a particular field, skills pertaining to ECG are forgotten. In routine practice, Internal Medicine, Cardiology, Emergency Medicine, Anaesthesia, and Critical Care specialists are the only specialists who remain well versed with ECG rhythm identification. Therefore, cases with any ECG related abnormalities are usually referred to them or are managed by them. During the COVID-19 pandemic, the number of these specialists were not enough to fight the battle and we needed to create an extra workforce to support them in patient care. Faculty, senior residents, junior residents from all specialities were put on floor duties.

To ensure that we do not compromise with patient care, the Advanced Centre of Continuous Professional Development (CPD) scheduled many training programmes in a short span of time for all involved in patient management at our Institute. During the preparedness training to combat COVID-19, one of the training sessions provided to all healthcare workers posted in designated COVID-19 wards was ECG rhythm identification and intervention for immediate management until experts could be consulted. A study done by Khan et al. on assessment of ECG and arrhythmia management in undergraduate students found that post-workshop assessments and feedbacks help in assessing any educational activity with precision [1]. This study was undertaken to evaluate the effectiveness of the training module in enabling participants with identification and immediate management of ECG rhythm abnormality in COVID-19 patients.

## Materials and methods

This was a cross-sectional study aimed to assess the effectiveness of interactive teaching sessions (physical/hybrid mode) for improvement in interpreting ECG and managing basic cardiac emergencies. It was conducted from April 2020 to February 2021 (10 months) through multiple training sessions, which included in-person and online live mode. Study participants included HCWs involved in patient care and management (faculty, senior residents, junior residents, and interns). This training on “ECG rhythm identification, interpretation, management in COVID-19 patients” was planned over three weeks by experts during which learning objectives of training, training module, assessment, and feedback were developed. CPD scheduled the program as a part of preparedness training to combat COVID-19. Participants were informed by CPD about their training dates and time through email. Participants were given the opportunity to attend the session, even if they did not wish to take part in the study. It was ensured by CPD to keep the batch strength for each training session to 30-35 participants. Ethical approval was obtained from the Institutional Ethics Committee, AII India Medical Sciences Rishikesh, India, via letter No. AIIMS/IEC/19/1035 and consent was taken from all the participants for the study.

Baseline data

At the time of registration for the session, participants were asked to fill out the online questionnaire and the consent form if they agreed to participate in the study. Baseline demographic characteristics included name, e-mail id, department, and present designation. Upon submission of the forms, each participant was given a unique identification number that was used throughout the study to ensure confidentiality and blinding of the researcher for data analysis. 

All participants attended the ECG training session. The session involved one hour of didactic lecture and one and half hours of interactive session during which case scenarios were discussed with the participants. The teaching material and questionnaire were prepared by two expert faculty of Internal Medicine and Cardiology and validated by four other faculty members. The sessions were delivered by the faculty of Internal Medicine, Cardiology, and Anaesthesia. Before delivering the session, these faculty underwent micro-teaching sessions and uniformity in teaching methods was ensured. Learning objectives were stated to participants prior to starting the training session. The participants undertook an online assessment (pre-test) which was made available to them on the day of the session via google forms just prior to training. The questionnaire was prepared by medical teachers (physicians and cardiologists). The questionnaire was aimed to determine participants competency in identifying, interpreting, and responding to basic cardiac emergencies related to rhythm abnormalities for timely intervention to save lives and prevent complications. The test comprised 15 multiple choice questions with one mark allotted for each correct response and zero for incorrect response. There was no negative marking. Participants were given 20 minutes to complete the 15-item questionnaire. Questions were mainly divided into four sections. There were four questions pertaining to normal ECG parameters, three questions on practical skills of doing an ECG, five questions in reference to the identification of various abnormal rhythms like atrial fibrillation and/or flutter, ventricular tachycardia and/or bradycardia, and various other blocks and the remaining three questions were about various drugs that could precipitate or treat cardiac abnormalities. There was no duplication of the questions and pre-test scores were not revealed to the participants. Scores below 50% (<8 out of 15 was considered unsatisfactory).

Participants were instructed to complete the second online assessment (post-test) after the completion of the session. The same questionnaire was used for pre and post-test. The study participants were instructed to fill the feedback questionnaires on a 5-Point Likert scale at the end of each training session. Feedback dealt with the effectiveness of trainers, methods used during the training, adequacy of interactive sessions, and utility of the course in real practice. 

Data entry and statistical analysis 

Data were compiled in Microsoft Excel (Microsoft Corp., Redmond, Washington). Results were subjected to statistical analysis using IBM SPSS Statistics for Windows, Version 24.0 (Released 2016. IBM Corp., Armonk, New York). Basic descriptive statistics defined baseline variables. Pre and post-test scores were compared by the difference in means using the paired t-test, p-value, and 95% confidence interval. A p-value of<0.05 was used to define the statistical significance.

Patient and public involvement

No patients were involved in the study. The participants in the study were healthcare professionals who were informed about the study design and were given the option of enrolling in the course even without giving consent for the study. Consent forms were mailed to all participants at the time of registration for the course along with the pre-test questionnaires. Those participants who gave consent for the study were given reminders for filling out pre-test questionnaires. Post-test questionnaires were mailed to all those who filled the pre-test questionnaires. Reminders were given to participants for filling out post-test questionnaires. All participants were mailed their pre and post-test scores and feedback was taken from them. 

## Results

In 10 months, 44 training sessions (23 in-person and 11 through an online platform) were conducted on ECG for cardiac rhythm identification, interpretation, and immediate management protocol. A total of 1057 doctors were trained during this 10-month period but only 800 participants consented to participate in the study. Only 682 of 800 participants who gave consent completed both pre and post-test questionnaires and were included in the final analysis. Participants were categorized as per the speciality and 318 (46.6%) belonged to medicine and allied departments, 231 (33.8%) to surgery and allied departments, and 41 (6%) were from para and pre-clinical branches. Participants were also classified as per their designation, which included 97 (14.2%) faculty, 184 (33.8%) senior residents, 313 (45.9%) junior residents, and 88 (12.9%) interns. 

**Figure 1:**
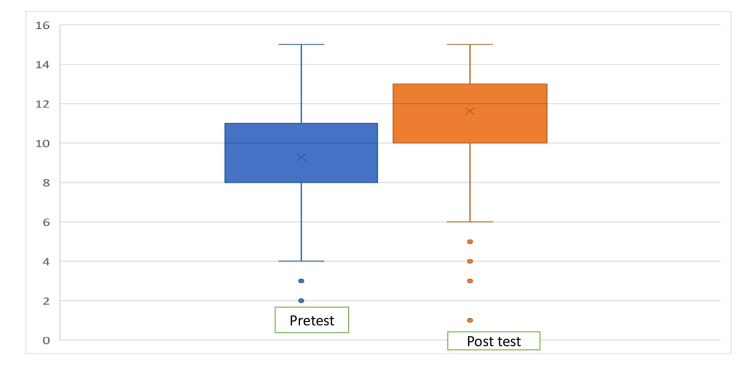
Boxplot graph displaying performance scores of participants in pre-test and post-test

Mean post-test scores showed a significant improvement in knowledge, which was found to be statistically significant (Table 1). The mean pre-test and post-test scores were 9.29/15 (61.9%) and 11.63/15 (77.5%), respectively, with a mean improvement of +2.34/15 (+15.6%). The extent of improvement varied considerably in post-test (Figure 1). The range of participant scores was the same in pre-test and post-test as the inter-quartiles range was 3 in both, but values of first and third quartile were higher in post-test scores of questions 1 and 3 (pre-test scores for items 1 and 3 were 8 and 11, respectively, and post-test scores were 10 and 13, respectively).

**Table 1:** Comparison of pre-test and post-test scores, n=682

Total score 15	Pre-test score	Post-test score	p-value
Range	2-15	3-15	<0.001
Mean	9.29	11.63	
SD	2.58	2.26	

A major proportion of participants obtained low pre-test scores (38.6%) while only 8.9% (n=61) attained unsatisfactory scores in post-test (Table 2). On doing sub-group analysis, it was found that participants from medicine and allied departments had higher mean baseline scores as compared to surgical and allied, paraclinical, and pre-clinical disciplines. However, each group showed good improvement in knowledge in the post-test that was found to be statistically significant (Table 3). Data analysis on pre-test showed that senior residents had the least and faculty had maximum baseline knowledge (Table 4).

**Table 2:** Satisfactory and unsatisfactory scores in pre-test and post-test, n=682

Total score 15	Pre-test, n=682	Post-test, n=682
Unsatisfactory (0-8)	263 (38.6%)	61(8.9%)
Satisfactory (9-12)	350 (51.2%)	382 (55.9%)
Outstanding (13-15)	71(10.4%)	239 (34.9%)

**Table 3:** Comparison of mean of pre-test and post-test scores of participants of various disciplines, n=682

Departments	Pre-test	Post-test	p-value
Medicine and allied	9.76+/- 2.59	11.82+/-2.01	<0.001
Surgery and allied	8.72+/-2.54	11.37+/-2.54	<0.001
Paraclinical and Preclinical	8.28+/-1.85	10.78+/-2.46	0.001

**Table 4:** Comparison of mean score of pre-test and post-test with respect to designations, n=682

Designation	Pre-test	Post-test	p-value
Faculty	9.67+/-2.55	12.03+/-1.94	<0.001
Senior resident	8.9+/-2.68	10.88+/-2.46	<0.001
Junior Resident	9.34+/-2.51	11.84+/-2.18	<0.001
Intern	9.49+/-2.58	12.11+/-1.73	<0.001

Only 184 participants submitted feedback after the session was over (Table 5). There was a positive response by participants on the effectiveness of skill delivery and adequacy of time for the training as most of the participants were in agreement. Of the respondents, 82% also agreed that the training would be useful in their clinical practice in handling patients in COVID-19 wards and 93.5% opined that instructors were effectively delivered in the sessions. 

**Table 5:** Feedback response of participants on 5-point Likert scale, n=184 AV: audio visual; COVID-19: coronavirus disease 2019

Parameter	Strongly Agree	Agree	Neutral	Disagree	Strongly Disagree
Effectiveness of Instructors	71	101	9	1	2
Adequate AV aids used	48	98	33	4	1
Adequate time for interactive lectures	58	104	16	5	1
Adequate and appropriate skill delivery	31	77	48	22	6
Adequate time given for training	50	89	24	17	4
Training will be useful in actual practice for COVID-19 patient care	49	102	22	9	2

## Discussion

Lavranos et al. showed that ECG is a very important bedside investigation that plays a vital role in the management of patients suffering from cardiovascular problems [2]. The number of drugs that are being used in critical care like antianginal, antiemetics, antibiotics, inotropes, bronchodilators, etc., can cause conduction abnormalities in patients [3]. As evident from various studies done by He et al., Bergamaschi et al., and Mengshi et al., COVID-19 and the drugs used for its treatment could result in cardiac rhythm abnormalities [4,5,6]; hence, there was an urgent need to train and re-train all clinicians and nurses in identifying rhythms and act in time to save patients. 

During the COVID-19 pandemic, not only medical specialists but also surgical, paraclinical, and preclinical departments got involved in patient care under the supervision of frontline clinical departments. Despite ECG teaching being a part of the undergraduate curriculum, these competencies fade away or weaken with time in students who take up surgical or other non-clinical streams. Identification of abnormal rhythm and its interpretation on ECG is a basic competency that physicians posted in critical areas/ non-critical areas should possess for monitoring and treating COVID patients. A low baseline knowledge among residents and pre-clinical departments in our study highlights the need for re-training or re-skilling on ECG interpretation to combat COVID-19 and comprehensive patient care.

Antiperovitch et al., Brooks-Harris et al., and Mahler et al. showed that case-based teaching, tutorials, interactive teaching sessions, and workshops are all effective modes of small group teaching and are being found useful and effective in teaching-learning methods [7,8,9]. In workshops, participants gain active experimental learning that is focused on specific learning objectives. Framing of learning objectives during the planning phase helped us in designing a focused as well as a concise training module, which was shared with participants during the training. This was one of the reasons for successful delivery and learning during the training sessions. During this pandemic, when everything has become digital, a workshop is being seen as an alternative method of teaching that can address the downfalls of self-directed and e-learning. The effectiveness of the ECG workshops has been trialled by Mahler et al. [9]. In this prospective randomised study with 223 participants, both workshop and lecture-based teaching formats showed a significant improvement in the test scores and were significantly higher than the SDL format. They showed that workshops enable interactions to occur between the teacher and the learner by encouraging active discussion among them [9]. In workshops, participants are empowered to integrate their theoretical knowledge with their practical skills. In our ECG workshops, participants focussed on practising ECG interpretation and then reflecting on their performance, which is not possible in lecture-based and passive teaching methods.

In the present training workshop, we have used an interactive workshop format to enable participants to use their knowledge to interpret ECGs and we examined the success of this educational approach. The workshop was unique and one of its kind in this pandemic time. It was like a pilot project to deal with the crisis. The effectiveness of this educational intervention was evaluated by studying the improvement in knowledge and skills and the results of our study reflect overall low baseline knowledge and good improvement in knowledge following the training session, which was found to be statistically significant. The duration of the training was deliberately kept less and it was free of charge for all participants. This was important because of the limited time that the HCWs could dedicate to a single component owing to their prior clinical responsibilities during the COVID-19 pandemic. To the best of our knowledge, this is the first large-scale study to examine the specific effectiveness of an interactive workshop for improving the ECG interpretative skills during the COVID-19 times. Assessment by multiple-choice questions plays a very important role in assessing competency-based learning [10,11]; hence, we conducted this study with pre and post-test multiple-choice questions (clinical vignettes), which were prepared by the expert panel of cardiologists and physicians. The objective assessment helped in assessing the participants immediately within a limited time. Clinical vignettes were used in the evaluation as they have been found to be helpful in assessing high-order thinking as shown in studies by Piryani et al. and Kish et al. [12,13]. Feedback was used to assess the efficacy of the training workshop in our study. Feedbacks provide a better understanding of any training workshop and help in improving the planning the future projects as was shown in a study by Newman et al. [14].

## Conclusions

During the COVID-19 pandemic, over 800 healthcare professionals from different fields of their expertise participated in this training programme, which was spread over 10 months using both physical and online sessions. The data revealed that healthcare professionals forgot the basic resuscitation techniques over time, which was revealed by the fact that junior healthcare professionals scored better than seniors in their pre-tests, even though changes in scores in the post-tests were significant in both groups. A low baseline knowledge in ECG interpreting skills reiterates the fact that a re-training/ re-skilling on cardiac rhythm identification and interpretation is necessary for all doctors involved in monitoring and treating COVID-19 patients. Interactive training (physical/hybrid model) is effective in improving ECG interpretative skills among doctors across disciplines and is the appropriate method to retrain/reskill them, especially for large-scale capacity building. This also highlights the need for further research to check for the effectiveness of these programmes in the long run. This study did not help to combat this pandemic directly but helped us to be better prepared. 
